# Paracellular and transcellular migration of metastatic cells through the cerebral endothelium

**DOI:** 10.1111/jcmm.14156

**Published:** 2019-02-02

**Authors:** Hildegard Herman, Csilla Fazakas, János Haskó, Kinga Molnár, Ádám Mészáros, Ádám Nyúl‐Tóth, Gábor Szabó, Ferenc Erdélyi, Aurel Ardelean, Anca Hermenean, István A. Krizbai, Imola Wilhelm

**Affiliations:** ^1^ Institute of Life Sciences Vasile Goldiş Western University of Arad Arad Romania; ^2^ Institute of Biophysics Biological Research Centre Hungarian Academy of Sciences Szeged Hungary; ^3^ Theoretical Medicine Doctoral School University of Szeged Szeged Hungary; ^4^ Doctoral School of Biology University of Szeged Szeged Hungary; ^5^ Medical Gene Technology Unit Institute of Experimental Medicine Hungarian Academy of Sciences Budapest Hungary; ^6^ Department of Physiology, Anatomy and Neuroscience Faculty of Science and Informatics University of Szeged Szeged Hungary

**Keywords:** blood‐brain barrier, brain metastasis, breast cancer, cerebral endothelial cell, incorporation, intercalation, melanoma, N‐cadherin, paracellular, transcellular

## Abstract

Breast cancer and melanoma are among the most frequent cancer types leading to brain metastases. Despite the unquestionable clinical significance, important aspects of the development of secondary tumours of the central nervous system are largely uncharacterized, including extravasation of metastatic cells through the blood‐brain barrier. By using transmission electron microscopy, here we followed interactions of cancer cells and brain endothelial cells during the adhesion, intercalation/incorporation and transendothelial migration steps. We observed that brain endothelial cells were actively involved in the initial phases of the extravasation by extending filopodia‐like membrane protrusions towards the tumour cells. Melanoma cells tended to intercalate between endothelial cells and to transmigrate by utilizing the paracellular route. On the other hand, breast cancer cells were frequently incorporated into the endothelium and were able to migrate through the transcellular way from the apical to the basolateral side of brain endothelial cells. When co‐culturing melanoma cells with cerebral endothelial cells, we observed N‐cadherin enrichment at melanoma‐melanoma and melanoma‐endothelial cell borders. However, for breast cancer cells N‐cadherin proved to be dispensable for the transendothelial migration both in vitro and in vivo. Our results indicate that breast cancer cells are more effective in the transcellular type of migration than melanoma cells.

## 1 INTRODUCTION

Brain metastases are devastating complications of lung or breast carcinoma, melanoma and other cancer types, characterized by challenging treatment options.^1^, ^2^ Melanoma is the third most common source of brain metastases,^3^ but has the highest risk to spread into the central nervous system (CNS).^4^ Brain metastatic lesions can be found in approximately three of four patients dying of melanoma.^5^ Breast cancer is the second most frequent cause of CNS metastases.^3^ Among different subtypes, triple negative (ie negative for estrogen and progesterone hormone receptors and also for Her2/human epidermal growth factor receptor 2) and Her2‐enriched (ie negative for hormone receptors and positive for Her2) mammary tumours are the most prone to give secondary brain tumours.^6^ Incidence proportion of brain metastases was found to be 11.37% and 11.45% in patients having metastatic triple negative or metastatic Her2‐enriched breast tumours, respectively.^7^

In brain metastatic melanoma, novel systemic targeted therapies and immunotherapies significantly increased the median overall survival of the patients; however, it is still below 2 years.^8^, ^9^ Among patients with breast cancer brain metastasis, the median overall survival time was reported to be 6 months for the triple negative subtype and 10 months for the Her2‐enriched subtype.^7^

Considering this poor prognosis, new treatment and prevention strategies are urgently needed,^10^ which imply understanding of the mechanisms involved. Development of metastases of the CNS depends on the unique interaction between tumour cells and cells of the neurovascular unit (NVU).^11^ It has been suggested that both blood‐brain barrier (BBB)‐forming tightly interconnected endothelial cells and glial cells have a Janus‐faced attitude towards cancer cells, that is killing the vast majority of brain invading metastatic cells, but protecting those which are able to overcome the detrimental mechanisms.^12^

Here we addressed the first unique step of brain metastasis formation, that is diapedesis of tumour cells through the cerebral endothelium. Interconnected by continuous tight junctions (TJs), brain endothelial cells form the tightest endothelial barrier in the organism^13^; therefore, extravasation of metastatic cells through this highly restrictive vasculature is a key step in the development of secondary brain tumours. After adhesion to the luminal surface of endothelial cells, cancer cells may incorporate into the endothelial monolayer,^14^ followed by the transmigration step.

Transendothelial migration of tumour cells might involve disruption of the TJs and consequently the paracellular movement of the tumour cells. We have previously demonstrated the ability of melanoma cells to breach the junctional complex of cerebral endothelial cells (CECs) through direct contact and secretion of soluble factors.^15^, ^16^ Not only TJs, but adherens junctions (AJs) might also be involved in this process, especially the transmembrane cadherin proteins. In non‐brain endothelial cells, N‐cadherin was observed to participate in the formation of heterocellular contacts between tumour cells and endothelial cells during transendothelial migration of melanoma cells.^17^ N‐cadherin is up‐regulated in response to transforming growth factor‐β (TGF‐β) released by metastatic cells, leading to an increase in the ability of melanoma cells to attach to and to migrate through CECs.^18^

Besides the paracellular route, brain‐invading cells might also take the transcellular way, through individual endothelial cells. Our previous results raised the possibility of transcellular migration of breast cancer cells^16^; however, no direct evidence of the movement of cancer cells through the cell body of CECs has existed so far.

By using high resolution transmission electron microscopy (TEM), here we aimed to investigate the transmigration routes of melanoma and triple negative breast cancer cells through brain endothelial cells. In addition, involvement of N‐cadherin in these processes was also assessed.

## 2 MATERIAL AND METHODS

### 2.1 Cell culture and in vitro models

A2058 human melanoma cells (obtained from the European Collection of Authenticated Cell Cultures) were maintained in Minimum Essential Medium (MEM) Eagle with Earle's salts and non‐essential amino acids (Sigma Aldrich) supplemented with 5% foetal bovine serum (FBS, Sigma Aldrich, St Louis, MI, USA) and Glutamax (Thermo Fischer Scientific, Waltham, MA, USA). B16/F10 murine melanoma cells were kept in Roswell Park Memorial Institute (RPMI) 1640 medium (Pan Biotech, Aidenbach, Germany) supplemented with 5% FBS (PAA Laboratories) and Glutamax. MDA‐MB‐231 human triple negative breast cancer cells were kept in Dulbecco's Modified Eagle's medium (DMEM) medium (Sigma Aldrich) supplemented with 5% FBS (Sigma Aldrich). Enhanced green fluorescent protein (EGFP)‐expressing MDA‐MB‐231 cells were obtained by transfection of the cells with the pEGFP‐C1 plasmid using TurboFect reagent (Thermo Fischer Scientific) and selection with G418 (Thermo Fischer Scientific). 4T1 mouse triple negative breast cancer cells were cultured in RPMI 1640 containing 5% FBS (PAA Laboratories) and Glutamax. TdTomato‐4T1 cells were obtained by transfection of the cells with the pcDNA3.1(+)/Luc2 = tdT plasmid using Lipofectamine 2000 (Thermo Fischer Scientific) and selected by single cell cloning after sorting with a BD FACSAria Fusion flow cytometer. For further selection, tdTomato‐4T1 cells were cultured in G418‐containing medium. EmGFP (emerald GFP)‐expressing 4T1 cells were prepared by retroviral transfection, as described elsewhere^19^ and selected on blasticidin S (Sigma Aldrich). The hCMEC/D3 human cerebral endothelial microvascular cells (abbreviated D3; obtained from Pierre‐Olivier Couraud, Institut Cochin, Paris, France) were grown on rat tail collagen‐coated dishes in Endothelial Cell Growth Medium‐2 (EGM‐2) Endothelial Bullet Kit including basal medium and supplements (Lonza) and 2.5% FBS (Sigma Aldrich). All cell lines were routinely tested for mycoplasma infection.

Primary rat brain endothelial cells (RBECs) were isolated from 2‐ to 3‐week‐old rats.^20^ After the removal of meninges, cerebral cortices were cut into small pieces and digested with 1 mg/mL collagenase type 2 (Sigma Aldrich) for 75 minutes at 37°C. After separation of myelin by centrifugation in 20% BSA, a second digestion was performed using 1 mg/mL collagenase/dispase (Roche) for 50 minutes at 37°C. Microvessel fragments were collected after centrifugation on Percoll (Sigma Aldrich) gradient (10 minutes on 1000*g*) and plated onto fibronectin/collagen‐coated dishes. Endothelial cells growing out of the microvessels were cultured in DMEM Nutrient F‐12 Ham (DMEM/F12, Thermo Fischer Scientific), 10% plasma‐derived serum (PDS, First Link), insulin‐transferrin‐sodium selenite (ITS) supplement (Sigma Aldrich), heparin (Sigma Aldrich) and basic fibroblast growth factor (bFGF, Sigma Aldrich). In the first 2 days, 4 μg/mL puromycin was added to the culture medium to remove contaminating cells.

Endothelial‐tumour cell co‐cultures were prepared as previously described.^15^, ^16^ Briefly, brain endothelial cells were cultured until confluence in filter inserts (Corning‐Costar Transwell Clear, Corning, NY, USA; for electron microscopy), microscope slides (ibidi, for immunofluorescence), culture dishes (for Western blot) or E‐plates (for impedance measurements). Tumour cells were seeded upon the endothelial monolayer in a density of 0.5‐1.5 × 10^5^ cells/cm^2^ surface and left for 5‐24 hours. CellTracker Red CMTPX (Thermo Fisher Scientific) staining was performed according to the manufacturer's instructions.

### 2.2 In vivo brain metastasis models

For transmission electron microscopy (TEM) studies, Balb/c mice were injected with EmGFP‐4T1 cells. For confocal microscopy, we used the FVB/Ant:TgCAG‐yfp_sb #27mouse line (obtained from the Institute of Experimental Medicine, Budapest, Hungary) expressing Venus‐YFP (yellow fluorescent protein) in endothelial cells. These animals were inoculated with tdTomato‐4T1 cells. All mice were housed and treated in accordance with widely accepted standards and the protocols were approved by the institutional care and the Regional Animal Health and Food Control Station of Csongrád County (permit numbers: XVI./2980/2012 and XVI./764/2018).

Animals were injected with 100 μL Ringer‐Hepes buffer containing 10^6^ tumour cells into the common carotid artery (for TEM) or 3 × 10^6^ tumour cells into the left ventricle (for immunofluorescence), under isoflurane anaesthesia. Animals were killed after 5 or 12 days, transcardially perfused with phosphate buffered saline (PBS, 10 mol L^−1^, pH = 7.4), then with 2.7% glutaraldehyde (Sigma Aldrich, for TEM) or 3% paraformaldehyde (for immunofluorescence) in PBS. Brains were removed and placed into the same fixative overnight at 4°C.

### 2.3 Preparation of ultrasections and transmission electron microscopy (TEM)

Whole brains were embedded in 10% gelatin and 100 μm sections were prepared using a Leica VT1000 S vibratome. Sections were examined under a fluorescence microscope. Sections containing EmGFP‐4T1 cells were further used.

The filter inserts or the selected brain slices were fixed for 2.5 hours in 2.7% glutaraldehyde and post‐fixed for 75 minutes in 2% osmium tetroxide. After dehydration in graded ethanol baths, the samples were immersed in graded ethanol‐Epon baths and then embedded in Epon 812. The blocks were cut with a Leica EM UC7 ultramicrotome, and the 50 nm thick sections were stained with uranyl acetate and lead citrate, then analysed with a Tecnai 12 Biotwin TEM.

### 2.4 Immunofluorescence and fluorescence microscopy

Cell cultures were fixed with 100% methanol at −20°C. After extensive washing in PBS, samples were permeabilized with 0.5% TritonX‐100 in PBS at room temperature and blocked with 3% bovine serum albumin (BSA) in PBS. The anti‐N‐cadherin antibody (BD Transduction Laboratories, Franklin Lakes, NJ, USA) was applied on the coverslips in a dilution of 1:100 in 1% BSA in PBS overnight. After three washings in PBS, coverslips were incubated with Alexa Fluor 488‐labelled anti‐mouse IgG secondary antibody (Jackson ImmunoResearch, Cambridgeshire, UK), dilution: 1:100. After three further washing steps, samples were mounted with FluoroMount‐G media (SouthernBiotech, Birmingham, AL, USA). Nuclear staining was performed with Hoechst 33342 (0.66 μg/mL) during the second washing step. Fluorescent signals were examined with a Nikon Eclipse TE2000U microscope.

Brain sections were prepared with a Reichert‐Jung Cryo microtome 1206. After post‐fixation, brains were cryoprotected in 30% sucrose (in 10 mmol L^−1^ PBS) at 4°C. Twenty micrometres of cryosections were prepared and used for immunofluorescence. Brain sections were placed in plates and subjected to antigen retrieval using 100% methanol for 30 minutes. Permeabilization was performed with 0.5% TritonX‐100 for 30 minutes at room temperature, followed by blocking with 3% BSA in PBS. The first antibody was applied in a dilution of 1:100 in 1% BSA overnight at 4°C. After washing in PBS, the secondary antibody (STAR RED anti‐mouse IgG; Abberior, Göttingen, Germany) was applied in a dilution of 1:500 in PBS for 1 hour at room temperature. After three further washing steps, samples were mounted with FluoroMount‐G media (SouthernBiotech). Nuclear staining was performed with Hoechst 33342 (0.66 μg/mL) during the second washing step. Fluorescent signals were examined with a Leica SP5 confocal laser scanning microscope.

### 2.5 Cell sorting and Western blot

EGFP‐MDA‐MB‐231 cells were co‐cultured with D3 cells for 24 hours. Cells were collected with accutase (Corning) and sorted with a BD FACSJazz stream‐in‐air cell sorter using the 488 nm laser. Mono‐cultured EGFP‐MDA‐MB‐231 cells or D3 cells were also collected with accutase, sorted and collected based on the gating parameters established when sorting the co‐cultured cells.

Cells were collected in radioimmunoprecipitation assay (RIPA) buffer. After the 30‐minute incubation on ice, cell lysates were centrifuged on 9500*g* for 30 minutes at 4°C. Protein concentration was determined with bicinchoninic acid (BCA) (Santa Cruz Biotechnology, Santa Cruz, CA, USA). Laemmli buffer was added to the samples followed by heating on 95°C for 3 minutes. Proteins were electrophoresed using standard denaturing SDS‐PAGE procedures and blotted on polyvinylidene difluoride (PVDF) or nitrocellulose (Bio‐Rad, Hercules, CA, USA) membranes. Afterwards, the non‐specific binding capacity of the membranes was blocked with 3% BSA or 5% non‐fat milk in TBS‐T (Tris‐buffered saline with 0.1% Tween‐20). Membranes were incubated with primary antibodies in TBS‐T using the following dilutions: 1:200 cofilin (Cell Signaling Technology, Danvers, MA, USA), 1:200 phospho‐cofilin (Cell Signaling Technology), 1:1000 β‐actin (Sigma Aldrich), 1:500 pan‐cytokeratin (Thermo Fischer Scientific), 1:250 claudin‐5 (Thermo Fischer Scientific) or 1:200 N‐cadherin (BD Transduction Laboratories). Blots were washed in TBS‐T and incubated with the secondary antibodies in TBS‐T, as follows: HRP‐conjugated anti‐rabbit IgG (1:1000, Cell Signalling Technology) or HRP‐conjugated anti‐mouse IgG (1:4000, BD Transduction Laboratories). After washing, immunoreaction was visualized using the Clarity Chemiluminescent Substrate (Bio‐Rad) in a ChemiDoc MP imaging system (Bio‐Rad). Image lab software version 5.2 (Bio‐Rad) was used for the quantification of the blots by densitometry.

### 2.6 Real‐time impedance monitoring

To monitor the effects of tumour cells on RBECs in real time, we measured the electrical impedance using the xCELLigence system following the manufacturer's instructions (Acea Biosciences). Briefly, cells were seeded in an E‐plate (ie, 96‐well tissue culture plates having micro‐electrodes integrated on the bottom) and allowed to attach onto the electrode surface over time. The electrical impedance was recorded every 30 minutes. When the impedance reached plateau (ie the monolayer reached confluence), the cells were treated overnight with 550 nmol L^−1^ hydrocortisone, 250 μmol L^−1^ CPT‐cAMP and 17.5 μmol L^−1^ RO‐201724 (Sigma Aldrich) to induce maturation of TJs. Tumour cells (2 × 10^4^) were seeded into the wells in a medium containing reduced serum levels (2.5%) and left for 8 hours. The cell impedance (which depends on cell number, degree of adhesion, spreading and proliferation of the cells and also the tightness of the junctions), expressed in arbitrary units (cell index) was automatically calculated by the software of the instrument.

## 3 RESULTS

### 3.1 Interactions of melanoma cells with brain endothelial cells in vitro

Since our previous results indicated that melanoma cells have increased ability to attach to and to migrate through brain endothelial cells than breast cancer cells, we aimed to investigate these phenomena at ultrastructural level.

**Figure 1:**
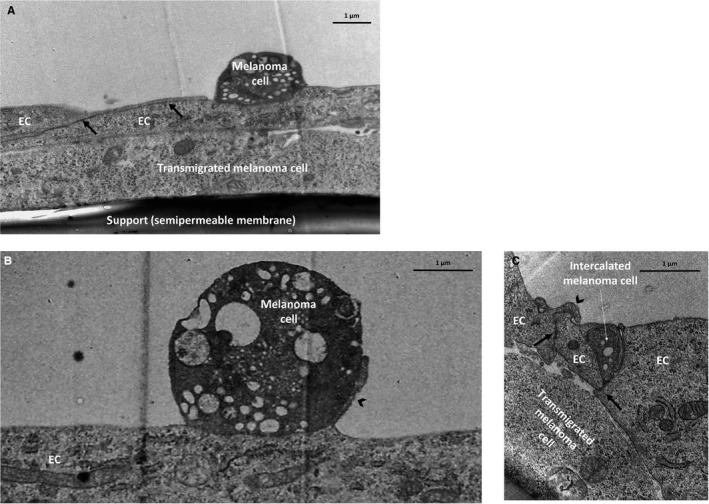
Adhesion of melanoma cells and intercalation between endothelial cells. B16/F10 melanoma cells were seeded on the top of confluent RBEC monolayers and left for 8 hours. Representative transmission electron micrographs show: a melanoma cell attached to brain endothelial cells in close proximity to the interendothelial junctions (A); a melanoma cell attached distant to the junctions (B) and a melanoma cell intercalated between endothelial cells (C). Arrows indicate interendothelial junctions. Arrowheads point to endothelial membrane protrusions. EC = endothelial cell

We first focused on the adhesion step, which precedes transmigration of tumour cells through endothelial cells. We observed several melanoma cells attached to brain endothelial cells in close proximity to the interendothelial junctions (Figure 1A), but also in regions distant from endothelial‐endothelial contacts (Figure 1B). Brain endothelial cells extended filopodia‐like membrane protrusions towards melanoma cells (Figure 1B), probably having an important role in the intercalation of the tumour cell between endothelial cells (Figure 1C).

**Figure 2:**
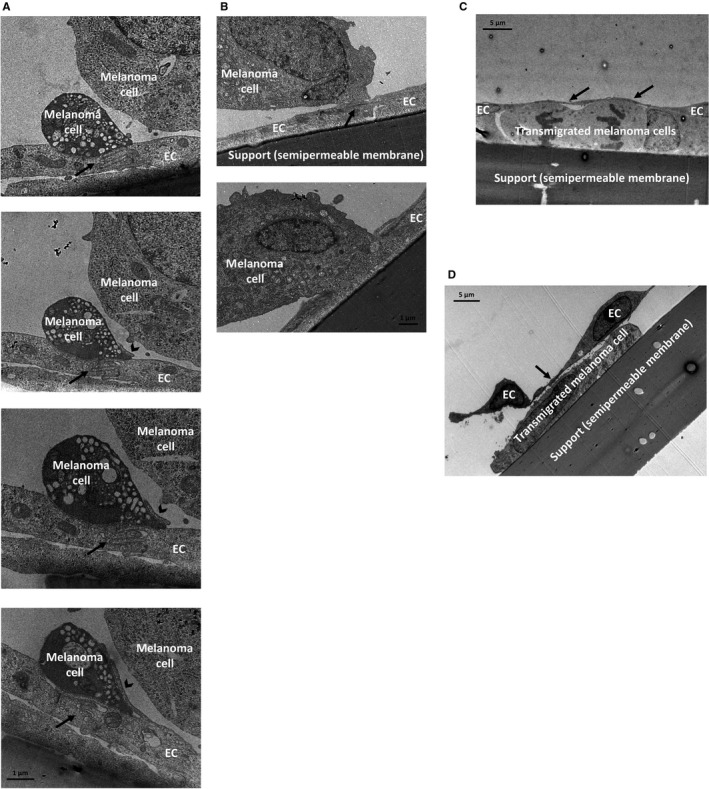
Transmigration of melanoma cells through brain endothelial layers. Melanoma cells (A, C: B16/F10; B, D: A2058) were seeded on the top of confluent RBEC monolayers and left for 8 hours. (A and B) Electron micrograph series of transmigrating melanoma cells. (C and D) Representative transmission micrographs of melanoma cells migrated through the brain endothelial monolayer. Arrow = interendothelial junction, arrowhead = membrane protrusion, EC = endothelial cell

As a result, melanoma cells transmigrated paracellularly, through the tight and adherens junctions between endothelial cells (Figure 2A and B). Some melanoma cells attached in clusters to the brain endothelial monolayer (Figure 2A) facilitating utilization of the same transmigration path by more cells, as we have previously shown.^15^, ^16^ We could also see transmigrated melanoma cells on the basolateral side of the endothelial cells. Transmigrated melanoma cells either moved further underneath the intact endothelial monolayer (Figure 2C) or, more often, were seen in the neighbourhood of the damaged endothelial cells (Figure 2D).

### 3.2 Interactions of breast cancer cells with brain endothelial cells in vitro

**Figure 3:**
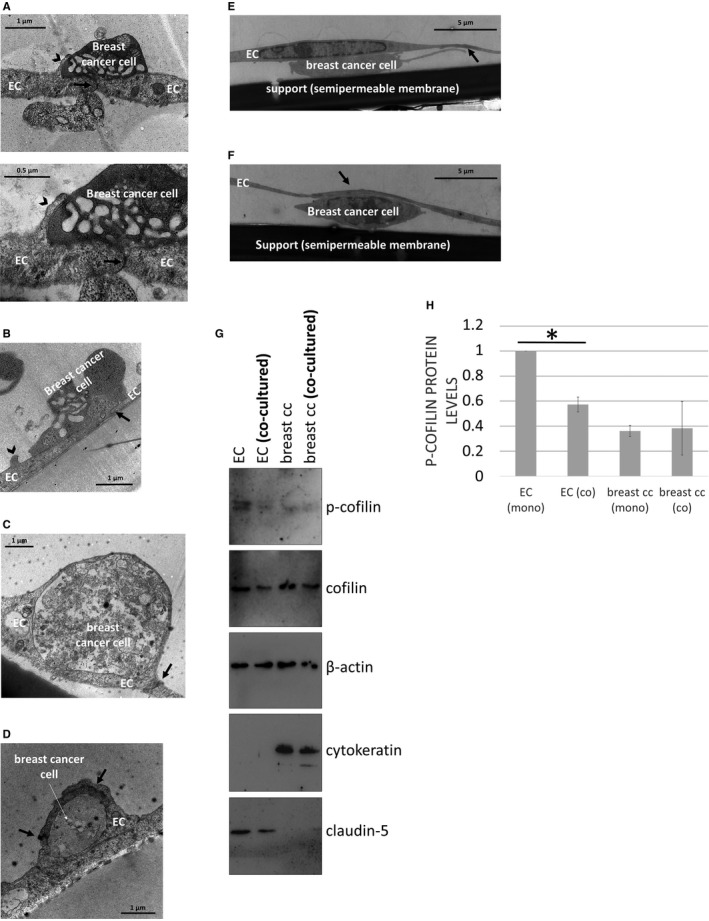
Interactions of breast cancer cells with brain endothelial cells. Breast cancer cells (A, C, E, F: MDA‐MB‐231; B, D: 4T1) were seeded on the top of confluent RBEC monolayers and left for 8 hours. Representative transmission electron micrographs show the adhesion (A, B) and incorporation (C, D) steps. (A) Bottom panel is a higher magnification of the image in the top panel. (E and F) Transmigrated breast cancer cells. Interendothelial junctions are indicated with black arrows, endothelial membrane protrusions are marked with black arrowheads. (G) EGFP‐MDA‐MB‐231 cells were co‐cultured with D3 cells. After 24 hours, the two cell types were separated by sorting. Representative Western blot images are shown. Purity of the samples is shown by the absence of epithelial‐specific cytokeratin in endothelial cells and absence of endothelial‐specific claudin‐5 in the tumour cells. (H) Quantification of p‐cofilin protein levels normalized to cofilin (average ± SD), based on the Western blot images. N = 3 independent experiments. *P < 0.01 as assessed by Student's *t* test (when endothelial cell—EC—mono and co‐cultures were compared)

Similar to melanoma cells, we could also identify breast cancer cells attached to cerebral endothelial junctions (Figure 3A and B), although less in number. In the proximity of these cells, filopodia‐like endothelial protrusions could be seen, similar to those observed in the vicinity of melanoma cells.

We have also detected several breast cancer cells completely covered by endothelial processes, incorporating the tumour cell into the monolayer (Figure 3C and D). Endothelial TJs were only observed on the top of or lateral to the mammary cancer cells, and were absent beneath the tumour cells, in the direction of migration. Similar integration of cancer cells into the brain endothelial layer was not observed for melanoma cells. Moreover, the endothelial monolayer remained almost perfectly intact despite the presence of transmigrated tumour cells (Figure 3E and F).

We have suggested that actin reorganization might play an important role in the changes observed in endothelial cells, including membrane protrusion formation, tumour cell incorporation and junctional disassembly. Recently, CECs were suggested to activate cofilin in response to extracellular vesicles secreted by breast cancer cells.^21^ To study the involvement of this signalling pathway in our system, we co‐cultured EGFP‐expressing MDA‐MB‐231 breast cancer cells with D3 brain endothelial cells. After 24 hours, we sorted the two cell types based on green fluorescence. Purity of the sorted samples was assessed by expression of cytokeratin in tumour cells, but not in endothelial cells and expression of claudin‐5 in endothelial cells, but not in tumour cells. Phospho‐cofilin was highly expressed in control brain endothelial cells, but significantly decreased in cells co‐cultured with breast cancer cells (Figure 3G and H), suggesting activation of cofilin signalling in CECs in the presence of breast cancer cells.

**Figure 4:**
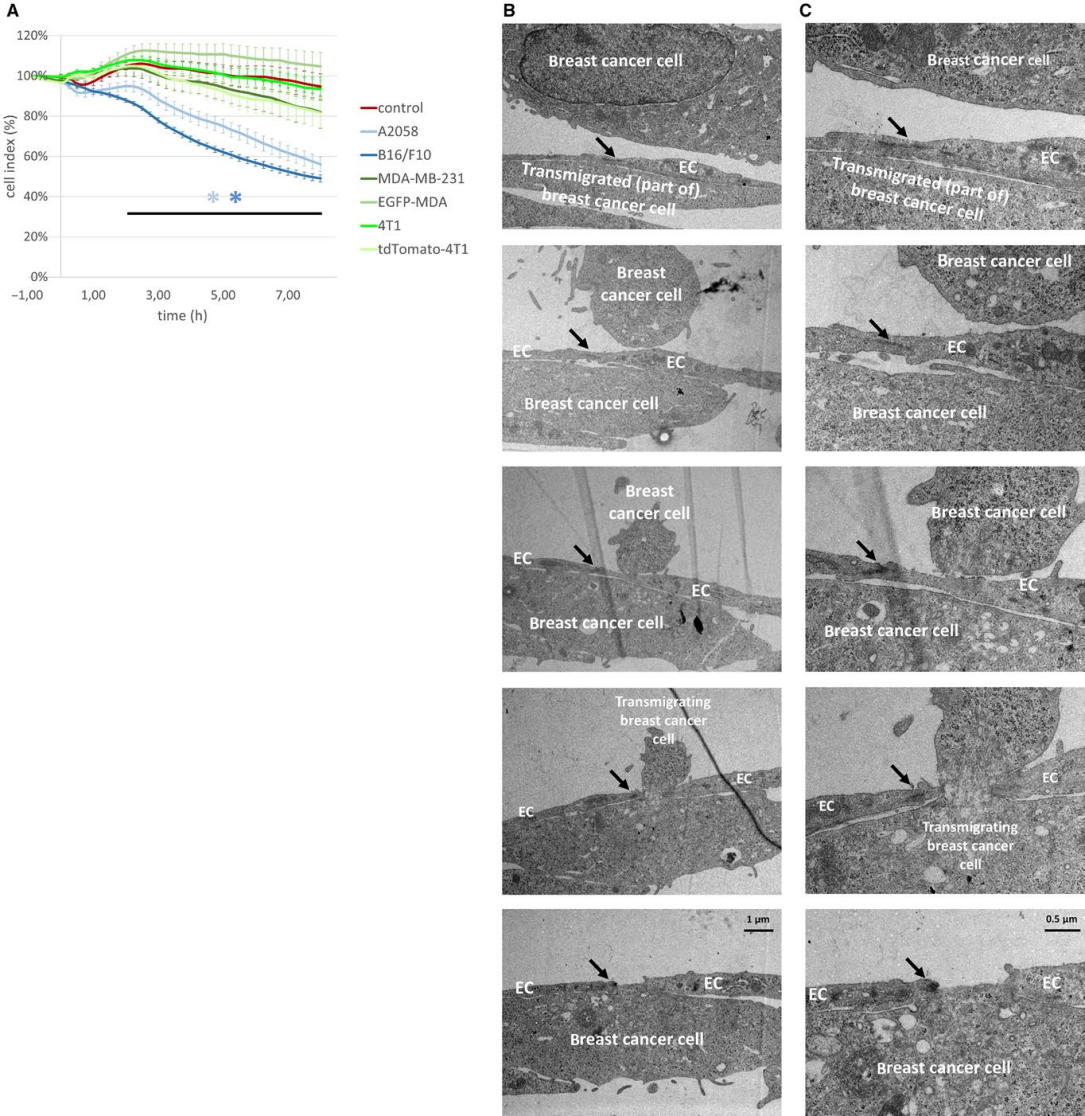
Transcellular migration of breast cancer cells through brain endothelial cells. (A) Tumour cells were seeded onto confluent monolayers of RBECs cultured in E‐plates and left for 8 hours. Impedance of the cells (represented by the cell index) was followed using the ACEA xCELLigence system. Results are expressed as % control and given as mean ± SEM. N = 4, *P < 0.01 (A2058 and B16/F10 melanoma cells compared to control) as assessed by ANOVA and Bonferroni's post‐hoc test. (B) MDA‐MB‐231 cells were seeded onto a confluent monolayer of RBECs and left for 8 hours. Images presented are electron micrograph series of a transmigrating breast cancer cell. Arrows indicate interendothelial junctions. (C) Higher magnification pictures of the respective images shown in (B). EC = endothelial cell

Previously, we have shown that melanoma cells, but not mammary carcinoma cells, can effectively disrupt cerebral endothelial TJs to migrate through the paracellular pathway.^15^, ^16^ In order to understand the effect of melanoma and breast cancer cells on the integrity of the brain endothelium, we followed the impedance of the monolayers after seeding different tumour cells upon them. Both B16/F10 and A2058 melanoma cells significantly decreased the impedance of brain endothelial cells already after 2 hours (Figure 4A). Neither of the four breast cancer cell lines had significant effect on the impedance. These data suggest that melanoma, but not breast cancer cells are able to significantly impair TJs of CECs. These results raised the possibility of transcellular migration of breast cancer cells.

Using electron microscopy, here we show for the first time that breast cancer cells are able to utilize the transcellular pathway—through individual endothelial cells—during their migration from the apical to the basolateral side of cerebral endothelial cells (Figure 4B and C).

### 3.3 Role of N‐cadherin in the transendothelial migration of tumour cells

**Figure 5:**
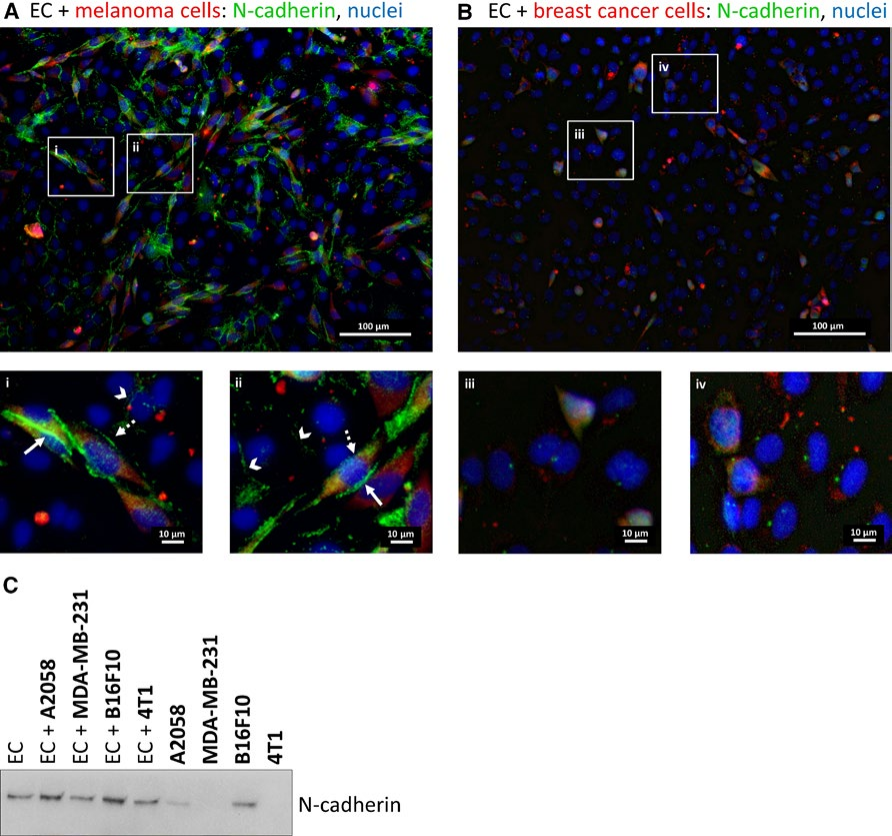
Role of N‐cadherin in the transendothelial migration of tumour cells in vitro. (A) CellTracker Red CMTPX‐stained A2058 melanoma cells were seeded onto a confluent monolayer of D3 cells and left for 5 hours. Representative immunofluorescence images are shown. The two bottom panels (i and ii) are higher magnifications of the respective sectors in the top image. (B) CellTracker Red CMTPX‐stained MDA‐MB‐231 cells were seeded onto a confluent monolayer of D3 cells and left for 5 hours. Representative immunofluorescence images are shown. The two bottom panels (iii and iv) are higher magnifications of the respective sectors in the top image. (C) MDA‐MB‐231 or 4T1 breast cancer or A2058 or B16/F10 melanoma cells were seeded onto confluent monolayers of RBECs and left for 5 hours. Protein samples were collected from mono‐cultures or co‐cultures (mixed cells). Representative Western blot shows expression of N‐cadherin in brain endothelial and melanoma cells, but not in breast cancer cells (MDA‐MB‐231 and 4T1). EC = endothelial cell

Besides disruption of TJs, melanoma cells must open the AJs of CECs during their paracellular migration from the apical to the basolateral side of the endothelium. N‐cadherin‐mediated interaction was shown to be involved in this process in non‐brain endothelial cells.^17^ Therefore, we investigated involvement of N‐cadherin in the migration of melanoma and breast cancer cells through CECs. When melanoma cells were seeded upon a confluent monolayer of CECs, tumour cells tended to rapidly intercalate among CECs. We observed the appearance of N‐cadherin in the melanoma‐melanoma and melanoma‐endothelial contact regions (Figure 5A). However, almost no N‐cadherin was detected in endothelial‐breast cancer cell co‐cultures (Figure 5B). Our Western blot results indicated that both A2058 and B16/F10 melanoma cells expressed high levels of N‐cadherin, but no N‐cadherin protein was detected in our breast cancer cell lines (Figure 5C).

**Figure 6:**
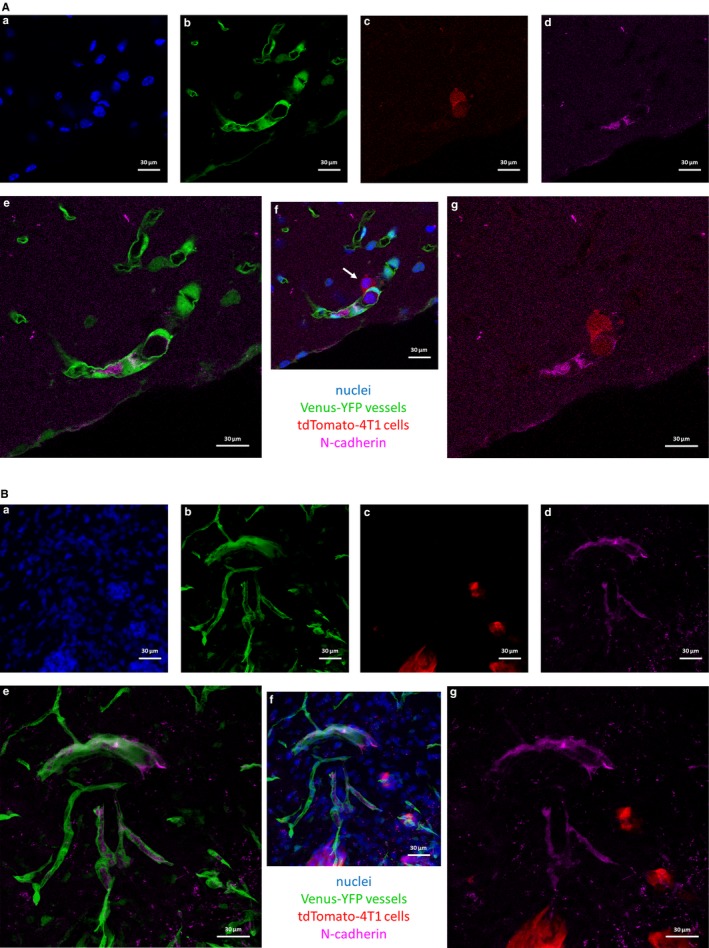
Role of N‐cadherin in the transendothelial migration of breast cancer cells in vivo. 4T1 mouse triple negative breast cancer cells expressing tdTomato red fluorescent protein were injected into mice expressing Venus‐YFP in endothelial cells. Mice were killed after 5 or 12 days (A and B, respectively). Representative confocal micrographs show that 4T1 breast cancer cells are N‐cadherin negative and metastasize efficiently to the brain. (A) Transmigrating cell in day 5 after inoculation of tumour cells (indicated by white arrow). (B) Already formed metastatic lesions in day 12. (a) nuclei (Hoechst 33342 staining). (b) endothelial cells (Venus‐YFP). (c) tdTomato‐4T1 cells. (d) N‐cadherin staining. (e) merged image of (b) and (d). (f) merged image of (a)‐(d). (g) merged image of (c) and (d)

Therefore, as a next step we investigated the ability of N‐cadherin‐negative breast cancer cells to give brain metastases in vivo. As a unique feature of brain metastasis formation, tumour cells arrested in cerebral capillaries survive for long time (approximately 3‐5 days) intravascularly before completing transmigration.^12^ Therefore, the first timepoint studied in the in vivo setup was day 5 after the injection of the tumour cells into the circulation of mice. At this timepoint, we observed transmigrating tumour cells which were all N‐cadherin negative (Figure 6A). On the other hand, expression of N‐cadherin was induced in the cerebral endothelium in the vicinity of some of the transmigrating cells (Figure 6A). By day 12 after the inoculation, several micro‐ and macrometastatic lesions were formed in the brain parenchyma. Importantly, 4T1 cells remained N‐cadherin negative throughout the metastatic process. N‐cadherin was only detected in some vascular segments in the endothelium of tumour cell‐bearing mice (Figure 6B). These results suggest that N‐cadherin is not necessarily needed by breast cancer cells to migrate through the brain vasculature and to form metastases in the CNS.

### 3.4 Interactions of breast cancer cells with the brain endothelium in vivo

**Figure 7:**
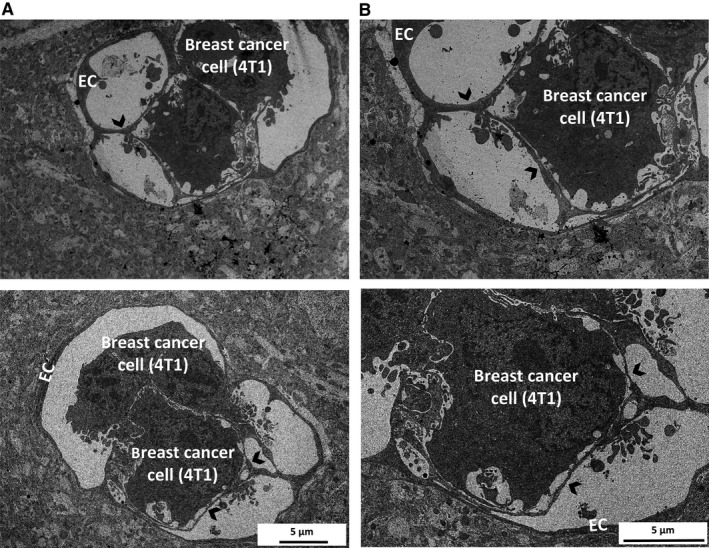
Interactions of breast cancer cells with the brain endothelium in vivo. EmGFP‐4T1 mouse breast cancer cells were injected into the circulation of mice. Ultrasections were prepared 5 days after the inoculation from brain tissue containing fluorescent tumour cells. Representative transmission electron micrographs show tumour cells surrounded by endothelial protrusions. Arrowheads indicate membrane protrusions. Images in (B) are higher magnifications of the respective images in (A). EC = endothelial cell

Finally, we examined interactions of metastatic breast cancer cells with the brain endothelium in vivo using TEM. In the brain sections obtained from mice injected with breast carcinoma cells, we observed active involvement of CECs in the metastatic extravasation process. Endothelial protrusions covering extravasating cancer cells were seen (Figure 7A and B).

## 4 DISCUSSION

Development of brain metastases is largely dependent on the ability of the tumour cells to migrate through the tightest endothelium of the organism, which forms the BBB. Involvement of CECs in extravasation of cancer cells into the CNS is largely uncharacterized and might be both offensive and defensive at the same time with the invading cells.^12^ By using TEM—a high resolution morphology technique—we assessed interactions of CECs with two of the most aggressive brain metastatic cells, that is melanoma and triple negative breast cancer cells.

Our in vitro and in vivo results indicate that CECs play an active role in the transendothelial migration of the tumour cells by extending filopodia‐like processes, which might guide invading cells towards low resistance points.^22^ Through this mechanism, endothelial cells may also incorporate the tumour cells or their extracellular vesicles, or isolate them from the circulating blood in vivo. Further studies are needed to understand whether this is a “friend or foe” reaction of endothelial cells, that is, whether endothelial protrusions facilitate transendothelial migration or engulf the tumour cells to protect the brain.

The observed ruffling of the endothelial plasma membrane is reminiscent of macropinocytosis,^23^ which is the entry route for platelet‐derived microparticles,^24^ pathogenic *Escherichia coli* bacteria 25 and nanoparticles 26 through the BBB. However, molecular mechanisms of tumour‐endothelial interactions still need to be studied. Cofilin activation indicates involvement of the actin‐myosin network. Nevertheless, cofilin‐induced modulation of actin dynamics in CECs has been shown to promote transendothelial migration of breast cancer cells and formation of brain metastases in vivo.^21^ According to the study of Tominaga *et al*,^21^ breast cancer‐derived extracellular vesicles containing miR‐181c promote destruction of BBB TJs through reorganization of actin, via down‐regulation of 3‐phosphoinositide‐dependent protein kinase‐1 (PDPK1) and down‐regulation of phospho‐cofilin (i.e. activation of cofilin). Therefore, remodelling of the endothelial cytoskeleton might be actively involved in regulating interactions of cancer cells with the cerebral endothelium. Nevertheless, the exact role of membrane and cytoskeletal changes of the cerebral endothelium in the metastatic process is far from being completely understood.

Among brain metastatic tumours, melanoma has the highest affinity towards the CNS. Earlier, this has been explained by the good capacity of melanoma cells to proliferate in the brain parenchyma.^27^ However, our previous results suggested that melanoma cells might also have an increased ability to migrate through cerebral endothelial layers in comparison to breast cancer cells.^16^ Especially, impairment of TJs of CECs was more pronounced in the presence of melanoma than mammary cancer cells.

Our present results are in line with these data, showing that melanoma cells can effectively use the paracellular route of transmigration. As a preceding step, melanoma cells intercalate between endothelial cells, which has previously been referred to as “incorporation”.^14^ However, based on the differences between diapedesis of melanoma and breast cancer cells through cerebral endothelial cells, presented here, we suggest using the term “intercalation” when tumour cells localize between two endothelial cells to proceed further to the paracellular transmigration. We use the term “incorporation” for describing tumour cells—independently whether intact or not—completely covered by endothelial cells. This phenomenon was mostly seen with breast cancer cells, most likely linked to the transcellular type of transendothelial migration.

To our best knowledge, we are the first to show direct evidence of transcellular migration of tumour cells through the BBB. The transcellular route of migration has initially been recognized for leukocytes,^28^ especially in the brain microvasculature.^29^, ^30^ As for tumour cells, the transcellular route of migration has only been described in the diapedesis of breast cancer cells through an in vitro vascular network 31 or through human umbilical cord endothelial cells (HUVECs).^32^ During this process an actomyosin transcellular circumferential invasion array is formed, regulated by myosin light chain kinase (MLCK) and myosin II regulatory light chain (RLC) phosphorylation. Further studies are needed to understand which signalling pathways are involved in the regulation of the cerebral endothelial cytoskeleton during transcellular migration of breast cancer cells.

During paracellular migration, melanoma cells disrupt the TJs of CECs.^15^ Not only TJs, but AJs are also involved in this process. Melanoma cells may adhere in clusters to cerebral endothelial cells, and attach to each other and to endothelial cells through N‐cadherin‐mediated junctions. As previously shown, the recruitment of N‐cadherin to heterocellular contacts plays an important role in the interaction of melanoma cells with non‐cerebral endothelial cells during transendothelial migration.^17^, ^33^ Moreover, after long‐lasting interactions, tumour cells are able to up‐regulate N‐cadherin expression in CECs during endothelial‐mesenchymal transition to enhance transendothelial migration.^18^ Here we show that N‐cadherin is mainly involved in melanoma‐endothelial interactions, but is dispensable in the transendothelial migration of breast cancer cells both in vitro and in vivo. A few days after the injection of breast cancer cells into the mice, we observed up‐regulation of N‐cadherin in brain microvascular endothelial cells; however, 4T1 breast cancer cells did not up‐regulate N‐cadherin expression either before, or during or after extravasation. This indicates an N‐cadherin‐independent transmigration of breast cancer cells in our in vitro and in vivo models, which might explain the lower transmigration ability of breast cancer cells in comparison to melanoma cells, as we have previously observed.^16^

Taken together, our results indicate that—through cytoskeletal and membrane reorganization—the microvascular endothelium is directly involved in extravasation of tumour cells into the brain. We also show that melanoma cells primarily utilize the paracellular route of transendothelial migration, while breast cancer cells are able to transcellularly migrate through the brain endothelial cell layer. During extravasation into the brain, triple negative breast cancer cells can migrate through the vessel walls in an N‐cadherin‐independent manner.

## CONFLICT OF INTEREST

None.

## AUTHORS’ CONTRIBUTION

H.H., C.F., J.H., K.M., Á.M. and Á.N‐T performed research; I.W. and I.A.K. designed and supervised the research study; G.S., F.E., A.A. and A.H. contributed essential reagents or tools; A.H., I.W., I.A.K. analysed the data; I.W. wrote the paper; all authors approved final version.
